# Comparison of optical microscopy and quantitative polymerase chain reaction for estimating parasitaemia in patients with kala-azar and modelling infectiousness to the vector *Lutzomyia longipalpis*

**DOI:** 10.1590/0074-02760160185

**Published:** 2016-07-18

**Authors:** Jailthon C Silva, Danielle A Zacarias, Vladimir C Silva, Nuno Rolão, Dorcas L Costa, Carlos HN Costa

**Affiliations:** 1 Universidade Federal do Piauí, Departamento de Medicina Comunitária, Instituto de Doenças Tropicais Natan Portella, Laboratório de Leishmanioses, Teresina, PI, Brasil; 2 Universidade Nova de Lisboa, Instituto de Higiene e Medicina Tropical, Lisboa, Portugal; 3 Universidade Federal do Piauí, Departamento Materno-Infantil, Teresina, PI, Brasil

**Keywords:** kala-azar, visceral leishmaniasis, parasitaemia, buffy coat, qPCR, microscopy

## Abstract

Currently, the only method for identifying infective hosts with *Leishmania infantum* to the vector *Lutzomyia longipalpis* is xenodiagnosis. More recently, quantitative polymerase chain reaction (qPCR) has been used to model human reservoir competence by assuming that detection of parasite DNA indicates the presence of viable parasites for infecting vectors. Since this assumption has not been proven, this study aimed to verify this hypothesis. The concentration of amastigotes in the peripheral blood of 30 patients with kala-azar was microscopically verified by leukoconcentration and was compared to qPCR estimates. Parasites were identified in 4.8 mL of peripheral blood from 67% of the patients, at a very low concentration (average 0.3 parasites/mL). However, qPCR showed 93% sensitivity and the estimated parasitaemia was over a thousand times greater, both in blood and plasma, with higher levels in plasma than in blood. Furthermore, the microscopic count of circulating parasites and the qPCR parasitaemia estimates were not mathematically compatible with the published proportions of infected sandflies in xenodiagnostic studies. These findings suggest that qPCR does not measure the concentration of circulating parasites, but rather measures DNA from other sites, and that blood might not be the main source of infection for vectors.

Protozoa belonging to the genus *Leishmania* are transmitted by sandfly bites, and lead to skin and mucous membrane ulcers, kala-azar, or visceral leishmaniasis (VL), which is a systemic and lethal disease. The species causing visceral disease, *L. infantum* and *L. donovani*, have evolved from those that cause cutaneous disease (Lukes et al. 2007). *L. infantum* is the causative agent of zoonotic VL, and infects several species of wild mammals, domestic dogs, and humans. *L. donovani* is transmitted only among humans and is restricted to the Indian Subcontinent and East Africa. In the vector, the parasites are flagellate (promastigotes), survive in the gut, and are transmitted by regurgitation as they invade the pharynx and proboscis (Killick-Kendrick et al. 1988). In vertebrate hosts, they survive in mononuclear phagocytes, without flagella (amastigotes). Parasites are distributed mainly in the spleen, bone marrow, liver, skin, mucosal epithelium and occasionally in the blood (Cecilio et al. 2014). Biting sand flies penetrate the superficial skin layers with their sharp mouthparts and feed upon the blood and skin-cell lysates that drain into their bite wound (pool-feeders). Therefore, they may be expected to ingest amastigotes from both the skin and blood (Lewis 1987).

Recently, quantitative polymerase chain reaction (qPCR) from blood was used to estimate the infectiousness of humans. The authors performed an experimental study with artificial xenodiagnoses to estimate the amount of circulating parasites necessary to infect sandflies, and followed it with a field study (Seblova et al. 2013, Miller et al. 2014). The underlying assumption was that transmission to sandflies originates from blood. However, the relationship of parasitaemia estimates by qPCR with actual parasitaemia (visualisation of amastigotes by microscopy) has not been determined. For this reason, we decided to compare parasitaemia of *L. infantum* as measured by direct microscopic examination with qPCR from whole blood of patients with kala-azar.

## MATERIALS AND METHODS

*Study population* - Thirty subjects were consecutively enrolled from January to July 2015, at the Natan Portella Tropical Diseases Institute, in Teresina, Brazil. All participants presented with clinical signs typical of VL. Diagnosis was confirmed by identifying parasites in a direct examination or culture of the bone marrow and by using the Kalazar Detect^TM^ Rapid Test (InBios International Inc., Seattle, WA, USA) immunochromatographic assay. Biological samples were collected before starting treatment. However, only patients with a positive parasitological result were enrolled in the study, irrespective of serology.

This study was part of a project named “Influence of the *Leishmania chagasi* genotype on leishmaniasis pathogenesis,” approved by the Ethics Committee in Research of the Piauí Federal University under the approval number 0116/2005. A free consent form was explained to and signed by all the participants or legal representatives.

*Microscopic examination* - Microscopy and DNA extraction were processed on the same day that blood was collected. Five millilitres of blood with EDTA was collected from each patient. About 200 μL were separated for DNA extraction. Plasma was separated by centrifugation at 1200 rotations per minute for 5 min and 20 μL of the buffy coat layer was aspirated and smeared on four slides. The slides were then dried and stained with Panoptic (Ranylab, Barbacena, Brazil). Each slide was fully examined thrice under a 100x objective with immersion oil. Fields containing amastigotes were photographed. Amastigotes were counted and registered according to their intracellular presence and the type of parasitised cell. The concentration of parasitised monocytes or neutrophils was calculated by dividing the number of parasitised monocytes or neutrophils by the total number of each lineage as determined by automatic blood counting at admission as part of the hospital’s diagnostic routine (Cell Dyn 3700 Diagnocel, Abbott Park, IL, USA). Slides were examined by an experienced scientist who continuously identifies amastigotes in bone marrow as part of the hospital routine (JCS). Additionally, a senior microscopist helped to narrow down this count.

Because amastigotes may be overlapped by host cell nuclei, some are difficult to detect due to the higher chromatographic density of the latter. Counts were adjusted to this loss, since the proportion of the nucleus to monocyte cytoplasm sizes vary from 2:1 to 1:1 (Wadsworth-Center 2015) and the neutrophil nucleus is about 40% of its cell size (Rosenbluth et al. 2006), the areas hidden by nuclei led to a loss of 1/2 to 2/3 of parasite counts in monocytes and 3/2 in neutrophils. Hence, amastigote counts in monocytes were multiplied by 2 and by 3 and those in neutrophils by 1.7.

*DNA extraction* - DNA extraction was performed with 200 µL of peripheral blood or plasma using the QIAamp DNA mini kit (Qiagen - Sample & Assay Technologies, Chatsworth, CA, USA) according to the manufacturer’s instructions. DNA concentration and purity were measured spectrophotometrically at 260 nm and 280 nm and 10 ng of DNA was used in each qPCR reaction.

*Generation of the standard curve* - A *L. infantum* isolate (50 µL) obtained from a patient from Teresina who was treated and cured, was inoculated in 1 mL of NNN (Novy-McNeal-Nicolle medium) and Schneider (Schneider’s Insect Medium, Sigma, St. Louis, USA) media, and the culture was incubated for five days at 27ºC under standard pressure conditions. On the fifth day, the parasites were counted in a Neubauer chamber. To extract DNA, 5 × 10^6^ parasites were centrifuged at 1200 *g* for 10 minutes. After centrifugation, the pellet was washed thrice in RPMI solution and immersed once in 200 µL of sterile saline solution, followed by DNA extraction according to manufacturer’s instructions for human blood and plasma. In order to generate the standard curve, DNA samples were adjusted to the equivalent of 2.5 × 10^4^ parasites/mL in pure water, followed by serial dilutions (10^4^, 10^3^, 10^2^, 10^1^ and 10^0^) using the kit’s elution buffer.

*qPCR* - Detection and quantification of *L. infantum* was performed through a series of qPCR reactions using the hydrolysis probes technology (Taqman®), as described by Rolão et al. (2004). Oligonucleotides and probes were designed based on the kinetoplast DNA minicircle sequence (kDNA) of an *L. infantum* isolate obtained from dog spleen in 1999. The sequences used for detection were Linf kDNA-F 5′-GGCGTTCTGCAAAATCGGAAAA-3′, Linf kDNA-R 5′-CCGATTTTTGGCATTTTTGGTCGAT-3′, and Linf kDNA_FAM-5′- TTTTGAACGGGATTTCTG-3′ (AF GenBank access number 169140). Reactions were performed at a final volume of 20 µL, with 4 µL of sample DNA (10 ng), 0.5 µL of custom probes (2.5 pmol) and primers (10.0 pmol) (Custom TaqMan^®^ Gene Expression Assays, Applied Biosystems, Foster City, CA, USA), 10 µL of 1x master mix (TaqMan^®^ Gene Expression Master Mix, Applied Biosystems, Foster City, CA, USA), and 5.5 µL of ultra-pure sterile water, in 48-well plates using the StepOne Real-Time PCR System (Applied Biosystems, Foster City, CA, USA). After initial denaturation for 10 min at 95ºC, the samples were subjected to 40 amplification cycles consisting of two steps: 15 sec at 95ºC and 1 min at 60ºC. The samples were assessed in triplicate. The standard curve was in duplicate, and two negative controls composed of the reaction mix with water instead of sample DNA, were added to each plate.

*Statistical analysis* - All collected data were imported from a Microsoft Excel 2003^®^ spreadsheet and statistical analysis was performed using Stata/SE^®^ 10.0 for Windows (College Station, Texas, USA). Means and 95% confidence intervals (95% CI) of normally distributed variables as well as medians were calculated. After normalisation by logarithmic transformation, parasitaemia was compared by blood cell type. Parasitaemia estimates in blood and plasma as obtained through qPCR were compared by the Student’s *t*-test. Spearman’s test was applied to verify the correlation between qPCR parasitaemia estimates.

*Transmission model* - In order to assess the biological significance of parasitaemia as measured by microscopy and by qPCR (e.g. whether the estimated parasitaemia was compatible with the already described proportions of infected sandflies when they feed on patients with kala-azar through xenodiagnoses), we estimated the probability of infection of *Lutzomyia longipalpis* if they had fed on the study population, by using a Poisson distribution model. For this task, the model’s parameters were: (i) measured parasitaemia by microscopy and by qPCR, (ii) the number of amastigotes necessary to infect one sandfly, and (iii) the volume of blood ingested by *Lu. longipalpis* in a single bite. It was assumed that for parameter (ii), at least a single parasite would be able to infect a sandfly (Seblova et al. 2013) and for parameter (iii), the volume of blood ingested by *Lu. longipalpis* was 0.4 μL (Ready 1978) as described by Miller et al. (2014).

## RESULTS

*Study population* - The study population was comprised of 19 males and 11 females with an average age of 12.1 years. Of the subjects, 17% were under four years of age, 53% were between four and 18 years of age, and 30% were over 18 years of age. The youngest subject was one year old, and the oldest was 23 years old. During hospitalisation, two patients deceased (7%) and two were admitted due to relapses. Three patients had an HIV-1 co-infection (10%). Parasites were detected by direct examination of the bone marrow in 27 patients (90%) and in 25 (83%) patients via culture.

**Fig. 1:**
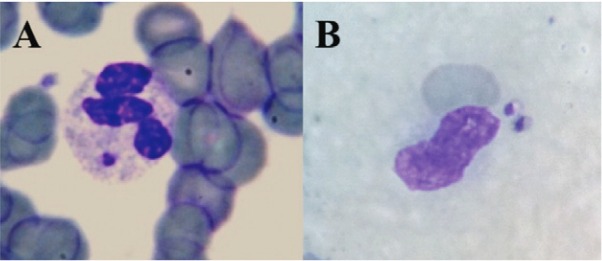
: aspect of detected amastigotes in neutrophils (A) and monocytes (B) from the peripheral blood of kala-azar patients. Note the presence of two amastigotes inside the monocyte.

*Parasitaemia measurement by microscopy* - Circulating amastigotes were observed in 20 out of 30 patients (67%, 95% CI: 47; 83). All the observed parasites were inside monocytes or neutrophils. Almost every time, a single parasite was found in each cell. Four patients had parasites in monocytes and neutrophils. At only three instances, two amastigotes were found inside monocytes from two patients (Fig. 1). Fifteen patients had parasites inside monocytes (50%, 95% CI: 31; 67) and nine patients had them inside neutrophils (30%, 95% CI: 15; 49). The mean parasitaemia obtained via microscopy was 0.3 parasites/mL (95% CI: 0.2; 0.5) and, when adjusted for unseen parasites hidden by the nuclei (multiplying by three), it was 1.0 parasite/mL. The mean number of parasitised monocytes by the total number of monocytes was 3.4/10^6^ and after adjustment, it was raised to 6.8 to 10.2/10^6^, but the median was zero [interquartile range (IQR): 0; 3.8]. This proportion was much lower among neutrophils, since only 0.4/10^6^ were parasitised (adjusted to 0.7/10^6^), and the median was also zero (IQR: 0; 0.7) (Table I). There was no correlation between the parasites inside monocytes with the parasites inside neutrophils.

**TABLE I:** Main results of the buffy coat microscopic examination for *Leishmania infantum* amastigotes in kala-azar patients

Characteristic	Proportion (%) (adjusted proportion)	Mean parasitaemia (adjusted concentration)	95% CI	Median (IQR)
Patients with infected amastigotes (%)	67	-	47; 83	-
Infected amastigotes/mL of blood	-	0.3 (0.6 - 0.9)^1^	0.17; 0.53	-
Monocytes with amastigotes/10^6^ monocytes	3.4 (6.8 - 10.2)^1^	-	-	0 (0; 3.8)
Neutrophils with amastigotes/10^6^ neutrophils	0.4 (0.7)^2^	--		0 (0; 0.7)

CI: confidence interval; IQR: interquartile range; ^1^proportion multiplied by two and three; ^2^multiplied by 1.7 (see text for details).

There was a statistically significant association between the proportion of patients with amastigotes in the peripheral blood and that of patients with a positive direct bone marrow examination (p = 0.03). However, this association was not observed when the presence of parasites in blood was tested separately with respect to monocytes or neutrophils. There was no association between the proportion of patients with circulating parasites and a positive bone marrow culture.

*qPCR blood and plasma parasitaemia estimates* - *Leishmania* DNA was detected in the blood of 26 out of 28 patients (93%, 95% CI: 76; 99) by qPCR and in the plasma of 16 out of 18 patients (89%, 95% CI: 65; 99).

Both patients who presented with negative results when tested in the blood also showed negative results in the plasma.

**Fig. 2:**
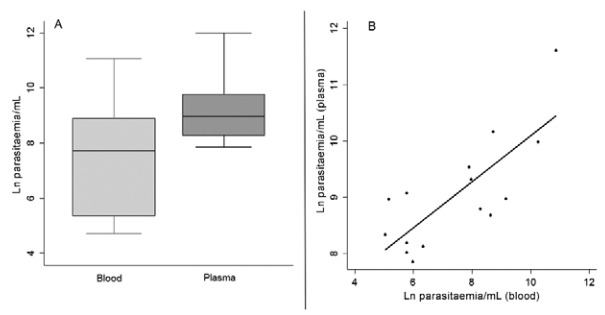
: comparison of parasitaemia estimates (or parasite equivalents) from blood and plasma from kala-azar patients (A) and correlation between both estimates with r = 0.73 and p*-*value < 0.001 (B).

The median of parasitaemia in blood as estimated by qPCR was 1219 parasite equivalents/mL (IQR: 191; 6666) and the mean was 7905 parasite equivalents/mL. In plasma, the median was 7188 parasite equivalents/mL (IQR: 3.388; 13,835) and the mean was 22,090 parasite equivalents/mL. When compared, the parasitaemia measured by plasma qPCR was statistically higher than that measured by blood qPCR (p < 0.001) (Fig. 2).

All three measurements, i.e. microscopy and qPCR from blood and plasma, were correlated. The weakest correlation coefficient was observed between microscopy and plasma qPCR (r = 0.46, p = 0.052). The correlation between microscopy and blood qPCR was moderate (r = 0.54, p = 0.03). There was an excellent correlation between both qPCRs (r = 0.73, p < 0.001) (Fig. 2).

*Prediction of Lu. longipalpis infectivity* - The probability of infecting one *Lu. longipalpis* if parasitaemia was estimated by microscopy was very low, with a median and mean of only 0.02% (IQR: 0; 0.02). After adjusting for parasites hidden by nuclei, the mean vector’s probability of infection only rose to 0.04%. However, when parasitaemia was estimated by blood qPCR, the vector’s probability of infection was high, with a median of 36.4% (IQR: 7.8; 92.9%) and a mean of 47.8% (Table II). In this scenario, 25% of the patients would infect over 90% of the insects (data not shown).

**TABLE II:** Prediction of infectivity of *Lutzomyia longipalpis* according to the parasitaemia measured by microscopy or quantitative polymerase chain reaction (qPCR) estimates from total blood, considering a volume of 0.4 μL ingested blood and that only a single amastigote could infect one female *Lu. longipalpis*

Method	Mean (% infected *Lu. longipalpis*)	Median (% infected *Lu. longipalpis*)	IQR (% infected *Lu. longipalpis*)
Microscopy	0.02	0.02	0; 0.02
Adjusted microscopy	0.04	0.03	0; 0.07
Total blood qPCR	47.8	36.4	7.8; 92.9

IQR: interquartile range.

## DISCUSSION

To our knowledge, this is the first attempt to compute the concentration of circulating amastigotes in the peripheral blood of humans with kala-azar and to model its significance in the transmission of kala-azar. Rohrs (1964) qualitatively described intracellular parasitism in peripheral blood and Chemli et al. (2006) counted circulating parasites in leukoconcentrates, but not the concentration of parasites in whole blood. With or without adjustment, blood concentration was extremely low, which explains the exceptionality of detecting circulating amastigotes in blood smears from kala-azar patients. Prior studies have shown higher leukoconcentration sensitivity than that described in this study (Chemli et al. 2006, Salam et al. 2012), possibly due to differences among study populations and *Leishmania* species. In any manner, the computation of leukocyte concentrations in peripheral blood for the microscopic diagnosis of kala-azar is promising and can be eventually explored. Observations on quantitative buffy coat (QBC^®^) showed that this task is reliable (Liarte et al. 2001).

Rohrs (1964) reported that parasites were seen only inside monocytes and neutrophils and that none of the cells was free. His observations were confirmed in this study, which also demonstrated that circulating intracellular parasitism is extremely low. The low number of amastigotes per cell suggests a recent cell infection, without sufficient time for parasite replication. Less competent phagocytes than macrophages, monocytes can be found in bone marrow; therefore, it is plausible that circulating parasitised monocytes originated in the bone marrow (Bosque et al. 1998, Levy et al. 2009). However, as recently demonstrated, the spleen is an important monocyte reservoir for other organs (Swirski et al. 2009) and it is not possible to decide where circulating amastigotes came from or where they would reside. On the other hand, parasitised neutrophils seem to have originated from the bone marrow not only for being found there, but also for being responsible for the earliest phagocytosis of parasites (el-Shoura & Sheikha 1991, Peters et al. 2008). Anyhow, it is reasonable to deduce that circulating amastigotes in the blood are an adaptation for dispersal and to occupy several tissues with parasitised resident macrophages, as in the liver, mucous membranes and the skin.

Parasitaemia estimated by qPCR in the blood was within the previously published limits (Mary et al. 2006). Although it presented a greater median than that observed in previous studies, the mean was lower. Although another study had a larger series of 696 patients that used by our laboratory (median = 388), it was inside the interquartile range and the mean was even lower (Zacarias D, unpublished data). Finally, the sensitivity of qPCR was excellent, indicating that qPCR could be used in the laboratory to diagnose kala-azar as a complement of serology, as was suggested for HIV-1 co-infected patients (Cota et al. 2013).

The extreme difference (over a thousand times) between parasitaemia medians measured by direct microscopy and those estimated through qPCR was unexpected. Additionally, the finding that estimated parasitaemia in the plasma was greater than that in the blood was also a surprise, since plasma is acellular. The finding of *L. infantum* DNA in the plasma suggests that parasitaemia estimated by qPCR of blood is exaggerated, due to the fact that the DNA detected in whole blood is not only from living organisms, but is mostly pure circulating DNA as demonstrated by qPCR from plasma.

The incoherence of qPCR-estimated parasitaemia with the reality of blood parasitism is highlighted by the routine white cell count. Indeed, parasitaemia in over 2000 parasites/mL, as estimated by qPCR, is about the same as that in cells such as basophiles, eosinophils, and monocytes and would be easily noticed in routine clinical analysis, which would not be true. Finally, these levels would not be compatible with that reported by studies with leukocyte concentrates, which point to a parasitaemia much lower than qPCR estimates (Dereure et al. 1998, Chemli et al. 2006). Indeed, it has been shown that DNA concentration was reduced drastically upon parasite death and disappeared completely after 48 h. For this reason, the excess of DNA measured by qPCR as compared to microscopy must be attributed to recently dead parasite DNA leached from other tissues (spleen, liver, bone marrow, skin) in “real time” (Prina et al. 2007). However, caution with the present results has to be highlighted since the qPCR technique was performed following older protocols. For instance, since the strain for generating the standard curve was not one that has already been typed and published but was an isolate from a local patient with kala-azar, the estimates of amastigote-equivalents might result in distinct values by other authors. Another point that should be stressed is that the most extreme values of plasma amastigote-equivalents were above the highest values of the standard curve. This might have led to imprecision and eventually to more extreme estimations. However, none of these caveats compromise the validity of the conclusions.

If this idea is proven true, extra attention must be paid when interpreting and publishing blood qPCR results, especially when transmission is assessed, since, as observed here, qPCR does not directly measure parasitaemia or infectivity, but rather measures the amount of circulating parasitic DNA. This molecular measurement would be, at most, a marker of the total amount of parasites in the organism, which is related to the amount of parasites available for vectors in the skin or blood. Indeed, this was verified in this study, and a moderate association of parasitaemia was obtained by microscopy and by qPCR. In fact, it is possible that this same association could be observed between skin and blood, in the same way that bone marrow parasitism was associated to blood qPCR (Verma et al. 2010). Therefore, the most appropriate term for blood qPCR results in patients with kala-azar would be “DNAemia”, or “total parasite load estimate”.

If the origin of the amastigotes transmitted to sandflies were blood, then the infectivity results obtained by previous xenodiagnostic studies would be compatible with blood amastigote counts. However, the studies performed in Brazil and Europe did not show such expected compatibility. Indeed, these observations of xenodiagnosis involving immunocompetent as well as immunosuppressed patients revealed large variation in the proportion of infective people and infected insects, albeit a higher proportion than that expected by parasitaemia as observed in this study. A Brazilian study described the proportion of humans with kala-azar but not with HIV-1 who infected *Lu. longipalpis* sandflies ranging from 25-28.5%, whereas the proportion of infected insects ranged from 2.5-14.8% (Deane & Deane 1955, Costa et al. 2000). Moreover, a Spanish study showed that all six patients with HIV-1 could infect *Phlebotomus perniciousus* in a proportion of 11-89% (Molina et al. 1994). Comparison between the observed and expected sandflies infected according to parasitaemia was then verified through mathematical modelling. The model considered three parameters: the estimated blood volume ingested by sandflies, the minimum limit of a single amastigote ingested by the insect at each meal to establish infection, and the observed parasite concentrations. It was then evident that the source of infection for vectors cannot be blood, since the proportion of infected *Lu. longipalpis* estimates and the concentrations obtained in microscopic examination, even after adjustment, are incompatible with the xenodiagnostic studies mentioned. Although the parasitaemia estimated through qPCR was within the range of what would be expected from the Spanish patients, the most extreme parasitaemia estimated by qPCR in a parcel of them suggests that almost all fed insects would become infected. These numbers are implausible even with the extreme situation of severely immunosuppressed patients with advanced AIDS described in Spain (Molina et al. 1999).

These findings lead to a final and decisive question: where do the parasites that actually infect the vectors come from? These data indicate that they do not originate from blood. On the other hand, the results indicate that they rather come from the skin, which is supported by the finding of a strong association of skin qPCR with the proportion of infected vectors in dogs (Courtenay et al. 2014). Since the *Leishmania* species that causes kala-azar evolved from a species that causes cutaneous leishmaniasis (Lukes et al. 2007) and the sand fly’s mouthparts are adapted to feed from the epidermis and the most superficial layers of the dermis (where blood vessels are scarce), it is most probable that the visceral leishmaniasis-causing species retained the original skin transmission form, and that the skin is the real source of parasites. In fact, an elegant study proved that parasites were present in the most superficial layers (Saridomichelakis et al. 2007) of dog epidermis and dermis. This idea is reinforced by the fact that skin supports the transmission of *L. donovani* through post-kala-azar dermal leishmaniasis lesions (Zijlstra et al. 2003). Unfortunately, there is still much to study regarding skin parasitism in patients with zoonotic kala-azar. If such studies are to be conducted, careful histopathological observations could reveal if skin is really the main source of infection for sand flies feeding from humans as suggested by this study.
